# Psychometric validation and interpretation of the Nocturia Impact Diary in a clinical trial setting

**DOI:** 10.1007/s11136-021-03060-4

**Published:** 2021-12-21

**Authors:** Stacie Hudgens, Amy Howerter, Ela Polek, Fredrik L. Andersson

**Affiliations:** 1 Clinical Outcomes Solutions, Tucson, AZ USA; 2 Clinical Outcomes Solutions, Folkestone, UK; 3 Ferring Pharmaceuticals A/S, Copenhagen, Denmark; 4 Clinical Outcomes Solutions, 1820 E River Rd, Ste 220, Tucson, AZ 85718 USA

**Keywords:** Anchor-based, Meaningful change threshold, NI Diary, Nocturia, Patient-reported outcomes, Quality of life

## Abstract

**Purpose:**

Psychometric evaluation of the Nocturia Impact (NI) Diary was conducted to support its use as a trial endpoint.

**Methods:**

As part of a randomized, controlled Phase 2 clinical trial investigating a novel drug candidate for nocturnal polyuria, adult nocturia patients completed the NI Diary and a voiding diary for three nights preceding their clinic visit at Baseline and Weeks 1, 4, 8, and 12 (end of treatment). Exit interviews were conducted to obtain patient impressions of the NI Diary.

**Results:**

A total of *N* = 302 participants were included. Confirmatory factor analysis (CFA) indicated that the 11-item measure is unidimensional with values of CFI, TLI, and RMSEA meeting relevant thresholds. Good internal consistency (Cronbach’s *α* 0.941) and test–retest reliability (intra-class correlation coefficients 0.730–0.880). Convergent validity with two reference measures was demonstrated with strong correlations of 0.573–0.730 were shown. Significant differences (*P* = 0.0018, standardized effect size = 0.372) between groups defined by number of night-time voids supported known-groups validity. Exit interviews in 66 patients indicated all participants experienced improvement in at least 1 NI Diary item and that a 1-point improvement on the item response scale and 1-void reduction per night (associated with an average best cut point on ROC analysis of − 11.6) constituted meaningful improvement. Anchor and distribution-based analyses identified a meaningful change threshold of − 15 to − 18 points on the NI Diary.

**Conclusion:**

The NI Diary is a reliable and valid patient-reported psychometric instrument which is fit-for-purpose to evaluate the impact of nocturia on patient quality of life in the clinical trial setting.

**Trial registration number and registration date** NCT03201419; June 28, 2017.

**Supplementary Information:**

The online version contains supplementary material available at 10.1007/s11136-021-03060-4.

## Plain English summary

Waking up in the middle of the night to urinate is a condition called nocturia and can be very bothersome. To get an idea of how much of an impact this condition has on a person’s life, we tested a NI Diary where patients with this condition can answer a series of 12 questions pertaining to how this condition affects them. During a 12-week clinical trial in which participants received a novel drug candidate, participants were asked on five occasions to answer these questions every evening for three consecutive nights preceding the visit to the clinic. A number of measurement tests were conducted on the diary to ensure it reliably assesses the severity of nocturia and its impact on quality of life (QoL) in the patient population. In exit interviews, participants expressed support for the usefulness of this Diary to reflect their views. This Diary may become a valuable tool for use in clinical trials and real-world studies.

## Introduction

**Fig. 1:**
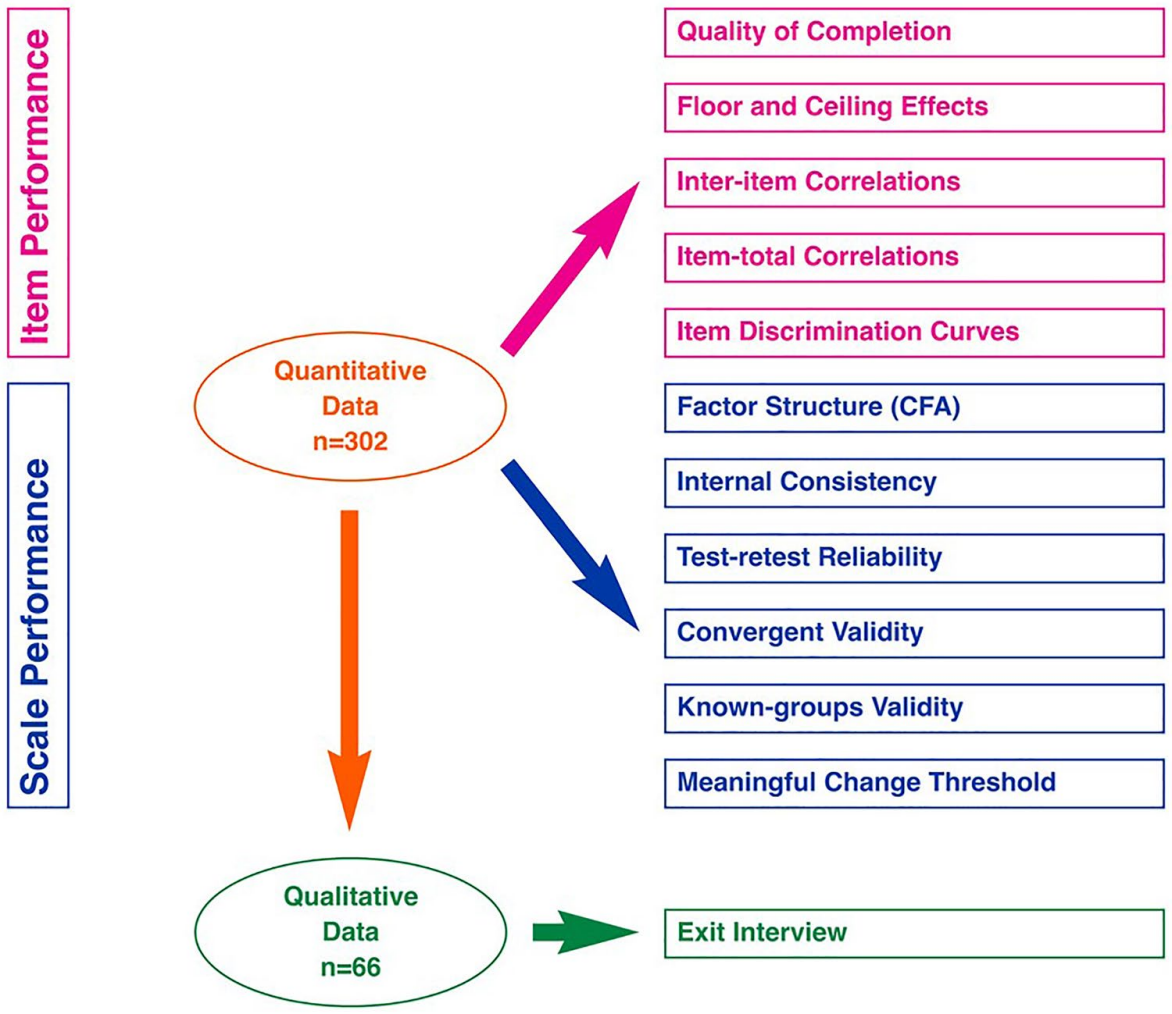
Overview of Psychometric Analyses performed for the current study

Nocturia, or waking to pass urine during the main sleep period [1], is a highly prevalent lower urinary tract syndrome affecting men and women of all ages, with higher rates in older populations [2, 3]. Although nocturia can have multiple causes, the most common is nocturnal polyuria—overproduction of urine at night [4]. Lifestyle modifications are the first intervention for the management of nocturia but as symptoms progress, such measures may be inadequate, and pharmacotherapy warranted [3, 5–10]. Nocturia has a pronounced negative impact on patient QoL [8, 11–13] and is associated with reduced work productivity, more frequent physician visits, socioeconomic burden [5, 6, 10, 12, 14], sleep impairment [15, 16], higher risk of falls and fractures, depression, and increased mortality [3, 17, 18]. There has, however, been an unmet need for a validated, reliable, and specific patient-reported instrument to assess the impact of nocturia on patient QoL. The most frequently used symptom-specific nocturia questionnaire, the Nocturia QoL (N-QoL) was validated only in males [19], with the content validity reexamined subsequently [20]. However, the measure did not meet the Food and Drug Administration (FDA) 2009 guidance [21] for content validity and the recall period (14 days or 1 month) was considered too long for a fluctuating disease [22]. To provide a more acceptable patient-reported outcome (PRO) measure for use in clinical trials, a 12-item Nocturia Impact (NI) Diary [22] was developed in dialogue with the FDA to measure the daily symptom impact of nocturia, to be used in conjunction with a nocturnal voiding diary. The NI Diary has 11 core items assessing impacts such as sleep disturbance, emotional disturbance, and fatigue, and a single overall QoL item. An earlier study with a small number of patients supported its psychometric properties [22]. The current study extends this work, investigating the reliability, validity, and interpretability of the NI Diary in a larger sample, using a range of evaluations (see Fig. 1).

## Methods

### Study design

A randomized, double-blind, placebo-controlled, multicenter Phase 2 clinical trial (NCT03201419; DAWN) [23] of patients with nocturia was conducted to investigate the safety and efficacy of a novel drug for nocturnal polyuria (Fig. S1 of the Online Resource). The current study is an independent, treatment-agnostic psychometric evaluation of the NI Diary performed to support the interpretation of the NI Diary as an endpoint in this trial. Patients completed the NI Diary and the nocturnal voiding diary for three nights preceding each visit at the clinic at Baseline, Week 1, 4, 8, and 12 (end of treatment).

### Study participants

Participants for this analysis were from the intent-to-treat (ITT) population from the trial and had completed the NI Diary at baseline. The sample size determination for the clinical trial was based on different dose–response scenarios indicating a required range of 60–75 patients per arm to achieve 80% to 90% power for the primary endpoint (reduction in nocturnal voids). The sample size of 302 patients exceeds the conservative minimum sample size of 10 patients per item for factor analysis [24], as well as providing sufficient power (80%) to detect, at two-sided *P* < .05, typical psychometric endpoints [25–29]. See the Online Resource for full details, including inclusion/exclusion criteria and ethics approval.

### Study instruments

#### NI Diary

The NI Diary© is a 12-item questionnaire with 11 core items and a single overall QoL impact question (Q12) that assesses the daily symptom impact of nocturia [22]. The NI Diary was completed in the evenings with the recall periods “thinking over the day” (items 1–6), “thinking about last night” (items 7–8) and “overall” (items 10–12). Each item is rated on a 5-point response scale from 0 to 5 (“not at all”; “slightly”; “moderately”; “quite a bit”; “a great deal”). Q12 of the NI Diary, evaluating the overall impact of nocturia, is used separately. The NI Diary total score, the sum of questions 1 to 11, has a range of 0 (lowest severity) to 44 (greatest severity). Both the total score and Q12 were transformed to a 0–100 scale. The total score was computed only if all items were answered, otherwise, it was defined as missing. Missing values were not imputed. For the purposes of this analysis, the total scores at each timepoint were averaged over the three nights, except for assessing quality of completion and confirmatory factor analysis (CFA).

#### Night-time voiding diary

The night-time voiding diary required participants to record the time of sleep, any awakenings for voiding, and the number of voids. The number of voids recorded over the three nights before the clinic visit was averaged to use in all reported analyses.

#### Patient Global Impression (PGI): Severity and Improvement

The PGI-Severity (PGI-S) is a patient rating of their current severity of nocturia reported as “none (1)”; “mild (2)”; “moderate (3)”; or “severe (4).” PGI-Improvement (PGI-I) provides a patient-rated summary of change in nocturia since starting study treatment reported as “very much better”; “much better”; “a little better”; “no change”; “a little worse”; “much worse”; or “very much worse”; coded 1 to 7, with higher scores reflecting poorer condition [30]. Full question details are provided in the Online Resource.

#### Exit interviews

Exit interviews in 66 patients were conducted by trained interviewers and consisted of 4 parts discussing: (1) experience of living with nocturia and its impacts; (2) the NI Diary and what constitutes meaningful change (assessed in terms of interpreting the response scale by item); (3) change in nocturnal voids and what constitutes meaningful change; (4) completion of PGI-S and PGI-I questions. Additional information, including sample size determination are in the Online Resource.

### Analytical methods

#### Quality of completion

The percentage of completion of the NI Diary items and the total score was described for the three nights preceding a clinic visit.

#### Item distribution and floor and ceiling effects

For each NI Diary item at each timepoint, the frequency and percentage of endorsements were presented for each response option. Floor effects (worst possible score on the scale) and/or ceiling effects (best possible score on the scale) were benchmarked at 20%.

#### Inter-item correlations and item-total correlations

Inter-item Spearman’s *ρ* correlations and corrected item-total polyserial correlations were calculated for NI Diary items at Baseline; the threshold of acceptable internal consistency set at ≥ 0.40 to ≤ 0.90 for inter-item correlations, and ≥ 0.40 for item-total correlations [31].

#### Item discrimination indices and curves

Item discrimination indices and curves were produced for each NI Diary item. The item discrimination index (calculation described in Online Resource) is a measure of how well an item differentiates between levels of severity, or in the case of the NI Diary, levels of impact. The discrimination index ranges from + 1 to − 1, with acceptable ranges > 0.60. The curves are presented for each response option with the percentage of participants choosing each option (*y*-axis) plotted against NI Diary total scores (*x*-axis).

#### Confirmatory factor analysis

CFA was conducted to test if the data support unidimensionality of the 11-item measure (item Q12, assessing the global QoL, is scored separately in accordance with the theoretical model) [32] Baseline data collected at Night 1 were used. CFA with weighted least square mean and variance estimators designed to handle ordinal data were computed and evaluated based on pre-defined thresholds considered to indicate close model fit: root mean square error of approximation (RMSEA) “poor” ≥ 0.113, “mediocre” = 0.094–0.113, “fair” = 0.066–0.094, “close” = 0.032–0.066, “excellent” ≤ 0.032 (because the RMSEA is interpreted as “the lower value, the better”, one only needs to consider the upper bound of the 90% CI); comparative fit index (CFI) of ≥ 0.95; Tucker–Lewis Index (TLI) of ≥ 0.95; and a standardized root mean residual (SRMR) of ≤ 0.08 [33]. Additionally, modification indices (MIs), quantified as the decrease in the *χ*^2^ value, indicated how model fit could be improved.

#### Internal consistency

Internal consistency reliability of the NI Diary (Cronbach’s *α* coefficient) was evaluated using Baseline data. Values > 0.70 are considered to be indicative of adequate internal consistency [34].

#### Test–retest reliability

Test–retest reliability was assessed using the Shrout–Fleiss intra-class correlation coefficient (ICC_2,1_) [35] (see Online Resource). An ICC of ≥ 0.70 is considered to be indicative of acceptable test–retest reliability [30, 36, 37]. Test–retest reliability was computed for the three sub-samples of patients showing little or no change between Baseline and Week 1 (see Online Resource).

#### Convergent validity

Convergent validity was assessed at Baseline in terms of Spearman’s correlations between the NI Diary and reference measures of the Insomnia Severity Index (ISI) [38] and bother of night-time urination frequency [39], with low convergent validity indicated if the coefficient is < 0.4, moderate if ≥ 0.4 to 0.7, and large if ≥ 0.7 [36, 37, 40]. Moderate-to-strong correlations between nocturia and sleep deficiency were hypothesized.

#### Known-groups validity

Construct validity was evaluated using the known-groups method. NI Diary scores at Baseline were compared among groups of participants differing on the number of nocturnal voids per night (0 to < 3 voids versus ≥ 3 voids) [40], using grouped *t*-tests. The extent of known-groups validity was considered by considering the extent or magnitude of the differences, using between-group effect size (ES) estimates, alongside the statistical significance of the difference in NI Diary mean scores (2-tailed *P-*value of < .05).

#### Interpretation of scores: meaningful change threshold (MCT)

The MCT on a PRO is the within-patient change in scores associated with what a patient perceives as a meaningful treatment benefit [41, 42]. The MCT was estimated using the pooled, treatment-agnostic, blinded data. Both distribution and anchor-based methods were used, with multiple anchor-based analytic methods utilized across five selected anchors (see Online Resource). As is standard practice [42], results were triangulated across the various methods, including the findings from the exit interviews, to arrive at an estimate(s) of MCT [43, 44].

#### Anchor-based methods

The change in the 11-item NI Diary score was calculated from Baseline to Week 12. Potential anchors, also measured as the change to Week 12, were: PGI-I, PGI-S, NI Diary Q12, the number of nocturnal voids, and PGI-I exit interview improvement [41]. Only anchors correlating with the change in NI Diary score above the 0.35 threshold were used in the analyses [44, 45]. A detailed description of change category derivation for each anchor is included in the Online Resource. Paired sample *t*-tests were used to evaluate the within-subject differences in NI Diary change scores between Baseline and Week 12 within each category [40, 43, 46], with the uncertainty in the estimate of mean change within each group captured by the 95% CI. The within-subject changes were expressed as standardized ES (SES) and interpreted based on Cohen’s recommendations: small change (SES = 0.20), moderate change (SES = 0.50), and large change (SES = 0.80) [45, 47].

#### Cumulative distribution function (CDF) curves

CDF curves of the change in NI Diary scores from Baseline to Week 12 presented NI Diary change within each anchor category. Absolute change from Baseline in NI Diary total score was expressed on the *x*-axis, and percentage of patients with a value at least equal to that value on the *y*-axis. Adequate separation between no change and “improved” categories was considered to indicate meaningfulness of the “improved” category.

#### Receiver operating characteristic (ROC) curves

ROC curves were an additional anchor-based approach used to determine the best cut point (BCP) in NI Diary change score (from Baseline to Week 12) for identifying participants who reported an average reduction of nocturnal voids of ≥ 0.5, ≥ 1, ≥ 1.5, and ≥ 2.5 during the 12-week period; the BCP was expected to increase the greater the number of nocturnal voids. The main criterion used to identify the BCP was the distance to the 0, 1 point (d(0,)), although an average across the cut points from three criteria (including sensitivity minus specificity and Youden’s Index) was also taken.

#### Distribution-based methods

A distribution-based approach for defining changes beyond measurement error was used to support the MCT estimated using the anchor-based approach. The estimated MCT must be greater than measurement error to rule out the possibility of participants being classified as a responder by chance [21, 42]. Distribution-based estimates were calculated as half the standard deviation (SD) at baseline and the standard error of measurement (SEM) (using Cronbach’s *α* as the reliability estimate), where SEM = SD √(1 − reliability) [48].

## Results

### Participant baseline demographics

Participant demographics are shown in Table 1. The mean age of participants was 58.8 years, and a higher proportion were women (60% female). Most participants were white (88%) and non-Hispanic (65%).

**Table 1:** Participant demographics

Demographic category	Study population *N* = 302	Exit interview population *N* = 66
Sex, *n* (%)		
Female	180 (59.6)	37 (56.1)
Male	122 (40.4)	29 (43.9)
Age (years)		
Mean (SD)	58.8 (12.82)	57.3 (13.17)
Median	60.5	57
Min, Max	50, 68	21, 84
Race, *n* (%)		
American Indian or Alaska Native	3 (1.0)	2 (3.0)
Asian	5 (1.7)	1 (1.5)
Black or African American	28 (9.3)	8 (12.1)
White	264 (87.4)	54 (81.8)
Unknown	2 (0.7)	1 (1.5)
Ethnicity, *n* (%)		
Hispanic or Latino	105 (34.8)	
Not Hispanic or Latino	195 (64.6)	
Unknown	2 (0.7)	

### Quality of completion

For all individual items no more than 11.8% of item responses were missing. Completion of all three diary nights was good at Baseline and Week 12 (*n* = 253/302 (84%) and *n* = 248/300 (83%), respectively). Few participants did not complete it at all (5 at Baseline and 4 at Week 12).

### Floor and ceiling effects

Floor and ceiling effects at Baseline, Week 1, and Week 12 are shown in Table 2.

**Table 2:** Floor and ceiling effects

	Baseline		Week 1		Week 12	
	Ceiling (%)	Floor (%)	Ceiling (%)	Floor (%)	Ceiling (%)	Floor (%)
Benchmark (%)	20	20	20	20	20	20
Item 1: Difficult to concentrate	13.5	4.1	24.8	2.4	47.2	0.4
Item 2: Low in energy and/or tired	6.4	10.5	15.7	4.2	29.2	0.4
Item 3: Unable to be productive or complete daily activities	14.5	5.7	30.4	2.8	49.8	0.7
Item 4: Avoid participating in activities	17.6	5.4	34.3	2.8	55.0	0.7
Item 5: Irritable or moody	17.9	6.4	32.2	2.1	49.4	1.8
Item 6: Limit your fluid intake	23.6	5.4	28.7	2.8	42.1	1.1
Item 7: Lay awake after using the bathroom at night	6.4	9.8	16.8	3.8	36.2	1.5
Item 8: Worried about tripping or falling	37.8	3.7	48.6	2.4	64.9	1.8
Item 9: Got too little sleep	3.7	16.2	14.0	5.9	34.7	2.2
Item 10: Worry that the nocturia will get worse	2.4	20.9	6.3	10.5	15.5	7.7
Item 11: Concerned with where the bathroom is	14.9	17.9	24.1	12.6	34.7	9.2
Item 12: Does nocturia presently impact your life?	2.4	21.6	7.3	11.5	19.2	6.6

Items 10 and 12 showed a floor effect at Baseline (> 20% of responses were in the category “A Great deal”). Ceiling effects were observed at the Baseline for Items and 8 with more than 20% of responses in the “Not At All” category and increased throughout the study, consistent with the expected improvement of nocturia symptoms

### Item-total correlations and inter-item correlations

Corrected polyserial item-total correlations for the NI Diary total score ranged from 0.607 to 0.841 indicating good internal consistency. Inter-item Spearman’s correlations ranged from 0.427 to 0.844 at Baseline, demonstrating that NI Diary items shared enough variance to be considered to measure the same latent concept (NI) yet, with the lack of perfect correlation, assessing different aspects of this concept.

### Item discrimination indices and curves

**Fig. 2:**
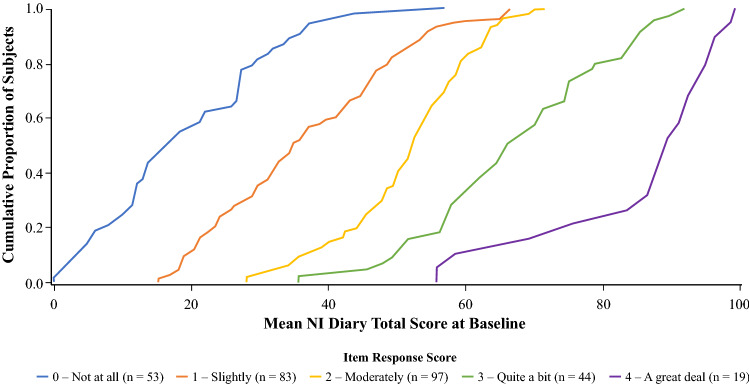
Item discrimination curve for all five response options for Item 5 (irritable or moody). Abscissa represents the Mean NI Diary total score at Baseline and ordinate represents Cumulative Proportion of Subjects. The curves for different response options are well-separated with direct correspondence between higher NI Diary scores below which all subjects score and higher severity of response of the item. For instance, for those responding 0 (Not at all) to indicate how much the experience moodiness or irritability, 100% of patients had a Baseline NI Diary score < 60 with most < 30, whereas for those who scored 4 (A great deal), almost all participants scored 60–100. *NI* nocturia impact

Discrimination indices for all items were close to or above the + 0.6 threshold, with a range of 0.535 (Item 6) to 0.915 (Item 9) indicating very good discrimination of all items. For most items, discrimination curves for all five response options differentiated well between different levels of severity (total scores). Figure 2 shows the Item 5 (*irritable or moody*) discrimination curve as an example; curves for other items are presented in Fig. S2 of the Online Resource.

### Confirmatory factor analysis

**Fig. 3:**
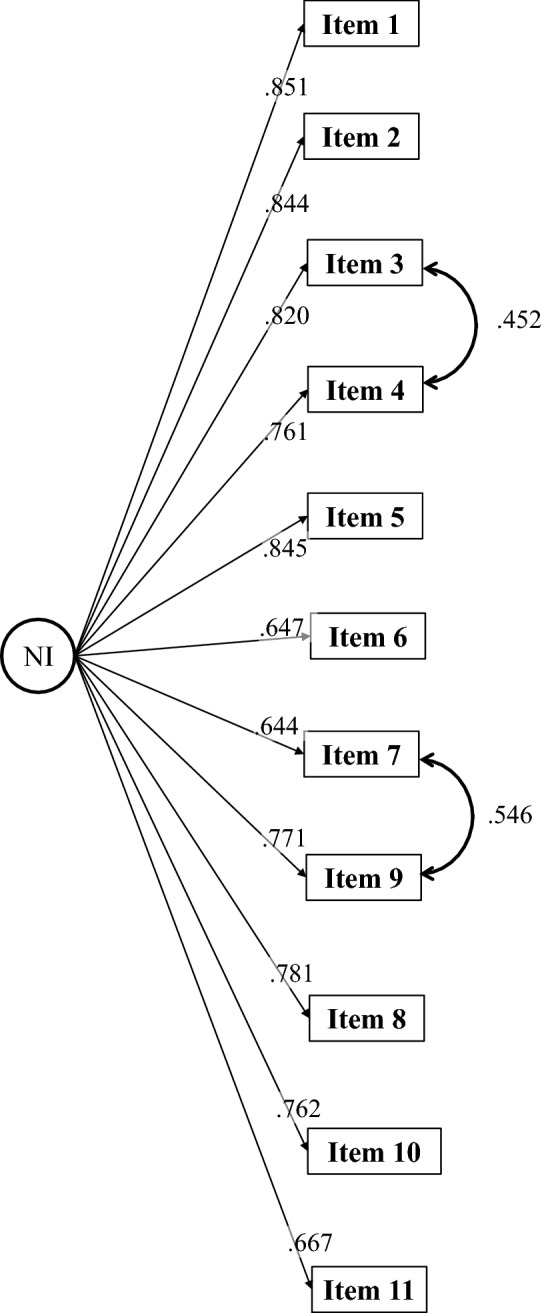
Confirmatory factor analysis: standardized factor loadings. *CFA* confirmatory factor analysis, *NI* nocturia impact

The initial model with 1–11 items showed modest fit (Table 3). MIs suggested adding residual correlations between items 4 (*avoided participating in activities*) and 3 (*unable to complete work and personal daily activities*) and items 7 (*lying awake after using the bathroom at night*) and 9 (*had too little sleep*). After this adjustment (see Fig. 3) the model with 1–11 items shows excellent CFI, TLI, and fair RMSEA (with upper CI bordering mediocre fit). The good fit of this unidimensional model provided an additional support to the theoretical assumption [22]) for scoring items 1–11 separate from the item 12 assessing global QoL.

**Table 3:** Fit indices for CFA model for NI Diary at Baseline (Night 1 data)

Baseline	*χ*^2^	df	*P*-value	CFI	TLI	RMSEA (90% CI)	SRMR	Range of standardized factor loadings
Unadjusted 11-item model	281.648	44	< .001	0.952	0.940	0.141 (0.125–0.157)	0.043	.64–.85
Adjusted 11-item model (with 2 residual correlations)	134.165	42	< .001	0.981	0.976	0.090 (0.073–0.107)	0.031	.64–.85

Fit indices were assessed as follows: RMSEA “poor” ≥ 0.113, “mediocre” = 0.094–0.113, “fair” = 0.066–0.094, “close” = 0.032–0.066, “excellent” ≤ 0.032; Acceptable: CFI of ≥ 0.95; TLI of ≥ 0.95; SRMR of ≤ 0.08

*CFA* confirmatory factor analysis, *CFI* comparative fit index, *CI* confidence interval, *RMSEA* root mean square error or approximation, *SRMR* standardized root mean residual, *TLI* Tucker–Lewis index

### Internal consistency

Cronbach’s *α* for the 11-item NI Diary was 0.941 notably greater than the 0.70 threshold. Additionally, the range of Cronbach’s *α* when a given item is removed ranged from 0.932 to 0.942 indicating that every item contributed to the high internal consistency.

### Test–retest reliability

The ICC (see Online Resource) for those who endorsed the “No change” response on the PGI-I at Week 1 (*n* = 33) was 0.880 [95% CI 0.777, 0.939]; for those who had no more than + / − 1 point of change between baseline and Week 1 on the PGI-S (*n* = 216) it was 0.730 [95% CI 0.661, 0.786]; and for those with no more than + / − 1 change in the average number of nocturnal voids between baseline and Week 1 (*n* = 125) it was 0.806 [95% CI 0.735, 0.859], indicating relatively high test–retest reliability of the NI Diary.

### Convergent validity

The baseline NI Diary demonstrated a high correlation with the baseline ISI (a measure assessing the severity of sleep-onset and sleep maintenance difficulties) [38] (Spearman’s *ρ* = 0.730), and a moderate correlation with the baseline bother rating of night-time urination frequency [39] (Spearman’s *ρ* = 0.587). The moderate-to-high correlation coefficients were as expected, confirming the convergent validity of the NI Diary.

### Known-groups validity

NI Diary mean scores were significantly higher in the group with ≥ 3 versus the group with 0–2 nocturnal voids (49.6 vs. 41.5, respectively; *P* = .0018), with the SES of − 0.37 indicating a difference of moderate magnitude, those with a higher number of voids reporting higher scores, i.e., impact, on the NI Diary.

## Interpretation of scores: MCT

### Exit interviews

Before entering the trial, more than half of participants (*n* = 39–62) reported experiencing each NI Diary item except item 8 ‘Worried about tripping or falling’ (*n* = 29). All participants reported improvement in at least one item of the NI Diary over the trial period. Fifty-three participants (80.3%) reported improvement in nocturnal urinations throughout the trial, none reported worsening, and 13 (19.7%) reported no change. Those reporting higher levels of improvement in the PGI-I experienced a greater reduction in nocturnal voids, with 81% of participants considering that a 1-point improvement on each NI Diary item response scale was meaningful. For instance, for the Tiredness question, one participant stated a 1-point difference means “Um, just that I'm getting more sleep and I'm not as tired”. A reduction of 1 void per night was considered to be meaningful (*n* = 30; 45.5%; see Online Resource Table S7). Across global rating responses (i.e., PGI-S, PGI-I) patients described the response categories to mean: “A little better” (sleeping more, less tired), “Much better” (Sleep more, less tired, mood improved, better concentration, work productivity better), and “Very much better” (Sleep more, less tired, mood improved, less impact on daily activities, better concentration, less avoidance of activities, easier falling back asleep, improved work productivity).

#### Correlation between the endpoint and anchors

The polyserial or Spearman’s correlation coefficients between change scores from Baseline to Week 12 for the NI Diary total score and the anchors were: (1) nocturnal voids, 0.389; (2) PGI-S, 0.669; (3) PGI-I, 0.639; (4) PGI-I Exit Interviews, 0.540; and (5) NI Diary Q12, 0.858, each greater than the benchmark value of 0.35 and thus all were used in the anchor-based analyses.

#### Anchor-based Analysis

For each of the anchors, monotonic improvements in the mean change in NI Diary total scores were generally observed for each level of categorical improvement on the anchor (see Tables S1. S2, S3, S4, S5 of the Online Resource). The SES of change in the NI Diary total score for each of the “1-category” (or equivalent) change groups was > 0.50 for each anchor, indicating at least a moderate degree of change in this group (Table 4).

**Table 4:** Within-subject change in NI Diary Total Score “No Change” and “1-Category”^a^ Improvement Anchor Groups (Extracted Tables S3, S4, S5, S6, S7)

Anchor	Change	*N*	Mean (SD)	Median	95% CI of Mean	*P*-value^b^	SES of Change^c^
Nocturnal Voids	> − 0.5 to < 0.5	21	− 8.0 (14.62)	− 1.5	(− 14.65, − 1.34)	0.021	− 0.55
	> − 1.5 to − 0.5	73	− 14.7 (21.22)	− 10.6	− 19.68, − 9.78	< .0001	− 0.69
	> − 2.5 to − 1.5	83	− 22.0 (24.22)	− 17.4	(− 27.31, − 16.74)	< .0001	− 0.91
PGI-S	No Change (0)	59	− 6.0 (13.91)	− 3.4	(− 9.65, − 2.40)	0.0015	− 0.43
	1-Point Improvement (− 1)	100	− 17.4 (18.61)	− 14.8	(− 21.13, − 13.74)	< .0001	− 0.94
PGI-I	No Change	33	− 1.5 (10.39)	− 0.8	(− 5.15, 2.22)	0.4227	− 0.14
	A Little Better	46	− 8.0 (15.34)	− 5.9	− 12.55, − 3.44	0.0010	− 0.52
	Much Better	74	− 20.4 (20.92)	− 16.7	(− 25.23, − 15.54)	< .0001	− 0.97
PGI-Interview	No Change	13	0.8 (10.85)	0	(− 5.80, 7.31)	0.8054	0.07
	A Little Better	10	− 8.2 (14.02)	− 5.9	− 18.21, 1.85	0.0981	− 0.58
	Much Better	17	− 21.9 (16.61)	− 21.2	(− 30.40, − 13.32)	< .0001	− 1.32
NI Diary Q12	No Change (0)	98	− 7.3 (11.32)	− 5.3	(− 9.55, − 5.01)	< .0001	− 0.64
	1-Category Improvement (− 1)	67	− 18.7 (13.81)	− 18.9	(− 22.06, − 15.33)	< .0001	− 1.35

*CI* confidence interval, *Max* maximum, *Min* minimum, *NI* nocturia impact, *PGI-I* Patient Global Impression-Improvement, *PGI-S* Patient Global Impression-Severity, *SD* standard deviation, *SES* standardized effect size

^a^1-Category represents the next level of improvement with non-overlapping CI with the “no change” group. For PGI-S and NI Diary Q12, this was 1-category within the respective scale. For nocturnal voids, PGI-I, PGI-Interview, this represents two categories of change in the respective scale, thus three levels are displayed in the table

^b^The *P*-value for each individual change group is derived from a paired (within samples) *t*-test assessing the difference over time

^c^Standardized Effect Sizes are calculated as the mean divided by the standard deviation. They are judged as: small = 0.20, moderate = 0.50, and large = 0.80

There was some degree of overlap in the 95% CIs for true mean change between the “1-category” and “no change” groups for the two anchors of change in nocturnal voids and PGI-I (the non-overlapping 95% CIs for the other anchors indicated that the groups were distinct). Consequently, both “1-category” and “2-category” change in these anchors were considered. These overlaps can, however, be explained by the “no change” nocturnal voids category including only 21 patients and the PGI-I anchors being limited by having no “moderately better” category.

The change in NI Diary total scores for the “1-category” change groups are summarized for each anchor in Table S6; the mean change scores range from − 8.0 (PGI-I) to − 18.7 (NI Diary Q12), and the median change scores from − 5.9 (PGI-I) to − 18.9 (NI Diary Q12). It is important to note that the exit interview patient reports of a reduction of 1 void per night being meaningful is consistent with the choice of the “1 category” − 0.5 to − 1.5 nocturnal void reduction category to indicate meaningful change, with a mean (median) NI Diary total score change of − 14.7 (− 10.6). The much larger mean (median) changes in the “Much better” category of − 20.4 (− 16.7) and − 21.9 (− 21.2) for the PGI-I and PGI-I Interview, respectively, versus those in the “A little better” category of − 8.0 (− 5.9) and − 8.2 (− 5.9), indicate that these values are likely to provide an overestimate of meaningful change. The mean NI Diary total score change across all 4 “A little better” and “Much better” PGI-I mean change values is − 15.0. The average 95% CI for true mean change across each anchor, within each “1-category” anchor change category, is − 8.0 to − 18.7.

#### Cumulative distribution function

A visual inspection of the CDF curves for each anchor revealed adequate separation between the “1-category” improvement category and the no change category for each anchor (Fig. S3 of the Online Resource), suggesting that the “1-category” improvement category is appropriate for assessing meaningful change. Maximum separation between the curves was achieved at NI Diary change scores of between approximately − 10 and − 20; generally, the median change within the “1-category” improvement group.

#### ROC analyses

The findings from the ROC analyses were consistent with those from the other anchor-based methods, with the BCPs increasing the greater the average reduction of nocturnal voids. The BCP at d(0, 1) in the NI Diary change score for identifying participants who reported an average reduction of nocturnal voids of ≥ 0.5 was − 6.82; for ≥ 1.0 it was -9.47; for ≥ 1.5 it was − 17.4; and for ≥ 2.5 it was − 24.2. Given that the patients in the exit interviews reported that a reduction of 1 nocturnal void was meaningful, the ROC curve for identifying participants who reported an average reduction of nocturnal voids of at least 1.0 [BCP =  − 9.47 for d(0, 1) and − 11.6 overall] is presented in Fig. S4 of the Online Resource.

#### Distribution-based methods

Using NI Diary total scores at Baseline, the 0.5 SD value was 10.90 and SEM 5.30, these providing lower bound estimates for the MCT.

#### Triangulation of results across anchor- and distribution-based data and exit interviews

The findings from the exit interviews indicated that a 1-point improvement in each NI Diary item is considered meaningful to patients; in the 11-item scale this would equate to an overall change of 11 points. This is consistent with the distribution-based estimates, with the value of 11 being larger than both 10.90 (0.5 SD) and 5.30 (SEM) and thus above measurement error. In the exit interviews the patients reported that an improvement of 1 void per night was meaningful; the ROC BCPs linked to this level of improvement were − 9.47 and − 11.6. The BCP from a ROC analysis would be expected to provide a lower bound for the MCT as it is the value that best distinguishes those who improve from those who do not. These findings suggest that a minimum MCT in the range of 10–11 points would be most likely to identify patients who have experienced a meaningful improvement in their symptoms. The anchor-based within-category change data support these findings with the average mean change across all anchors in the “1-category” improvement group of − 14.0 points, ranging from − 8.0 in the “A little Better” PGI-I category to − 18.7 in the NI Diary Q12 (and the average 95% CI also being − 8.0 to − 18.7). Taking into account the maximum separation observed in the CDF curves between − 10 and − 20 and the non-overlapping CIs for the “no-change” and “1-category” improvement groups, a conservative reduction of 15 to 18 points was taken as the MCT (in line with the smallest median change score in the non-overlapping groups of − 14.8). Thus, taking a reduction of 15–18 points in the NI Diary total score as the MCT would be consistent with all the results presented, anchor- and distribution-based as well as the patient perspective provided in the exit interviews.

## Discussion

This study has provided additional psychometric evidence to support the validity and reliability of the NI Diary, together with an estimate of meaningful change, thus enhancing the interpretation of improvement on the NI Diary. The CFA supported the hypothesized unidimensionality of the 11-item NI Diary and the scoring algorithm. This was further evidenced by high internal consistency reliability of the measure and with inter-item correlations in the range 0.40–0.90 indicating that items were generally not redundant or overlapping. Item discrimination curves indicated response categories were adequately separated. A proposed MCT in the range of 15‒18 points for the standardized NI Diary total score was determined by triangulating information from the within-category change for all five anchors with the findings from the ROC analysis and distribution-based methods, together with findings from the exit interviews, and provides a conservative estimate of meaningful change.

All analyses were conducted following the FDA Guidance for development and validation of patient outcomes [21]. However, a few limitations exist for the analyses presented. Incorporating post hoc correlated residuals in the CFA model (justified by similar item wording), nearly always improved model fit, but at the possible expense of generalizability of the model and with implications for the equal weighting of items within a sum score [49, 50]. When models are modified based on MIs (which often can be unstable), cross-validation of results is highly recommended in another sample to test validity of the modified model) [51, 52]. The limitation in this study stems from the lack of such cross-validation using a different sample. Within the Exit Interview, what constitutes meaningful change was only queried for the NI Diary and nocturnal voids, thus no claims about meaningfulness of change from the patient’s perspective can be made for the PGI-S or PGI-I categories of change, although those scales were debriefed with patients in work preceding the inclusion in the clinical trial.

Determining what constitutes a meaningful change on an instrument requires linking meaningfulness from the patient’s perspective with statistical determination of response thresholds that may be interpreted as a treatment benefit. This is the first psychometric validation and examination of response thresholds for the NI Diary using a mixed methods approach with clinical trial data. While there are benefits of applying multiple anchors and multiple analytic methods, there are no clear and concise guidelines for how to interpret these results and determine a threshold, especially if threshold values vary between anchors. Moreover, the thresholds are sample dependent and thus require further validation using comparable datasets.

Despite these limitations, this research presents parameters for interpreting the scores in the nocturia patient population. Exit interviews demonstrated that patient impressions on the NI Diary were in alignment with quantitative psychometric data, thus providing support for the use of NI Diary in both clinical trial and real-world studies. Overall, these findings provide substantive evidence that the NI Diary is fit-for-purpose for deriving patient-relevant endpoints in clinical research for nocturia.

## Supplementary Information

Below is the link to the electronic supplementary material.

Supplementary file1 (DOCX 187 kb)

## Data Availability

No.

## Code availability

N/A.
