# Supplementary material for: Psychometric validation and interpretation of the Nocturia Impact Diary in a clinical trial setting
Source: Qual Life Res. 2021 Dec 21;31(6):1837–48. doi: 10.1007/s11136-021-03060-4 (PMC9098619; doi:10.1007/s11136-021-03060-4)
Supplement: Supplementary file 1 — Supplementary file1 (DOCX 187 kb) [file 11136_2021_3060_MOESM1_ESM.docx]

**Supplementary Material to Hudgens et al., Psychometric Validation and Interpretation of the Nocturia Impact Diary in a Clinical Trial Setting; Quality of Life Research**

**Corresponding Author: Stacie Hudgens, Clinical Outcomes Solutions, Tucson, AZ 85718, USA; Phone: 1-520-325-9710; email:** [**stacie.hudgens@clinoutsolutions.com**](mailto:Stacie.hudgens@clinoutsolutions.com)

**Figure S1. Clinical Trial Schematic**

Abbreviations: R = randomization; V = visit

Note: After Visit 1, the diary was completed by the patient at home for 3 consecutive days before the next visit.

Baseline=Visit 4 (Randomization), Week 1=Visit 5 (not depicted in the picture), Week 12=Visit 8

**Ethics Approval**

Ethics approval for the DAWN trial was obtained by Institutional Review Boards (IRBs), Independent Ethics Committees (IECs) or local Research Ethics Boards in each country/region where the trial was conducted. The leading national IRB/IECs were Belgium: UZ Leuven; Canada/USA: Quorum Review (File # 32413); Czechia: Multicentricka Eticka Komise; Germany: Landesärztekammer Baden-Württemberg Ethikkommission; Hungary: Egeszsegugyi Tudomanyos Tanacs Klinikai Farmakologiai Etikai Bizottsaga; Poland: Komisja Bioetyczna.

**Power Analysis**

**Psychometric Tests**

Post-hoc power calculations indicated sufficient power (80%) to detect, at two-sided p<0.05, differences between two study groups of equal size (n=150) equivalent to a small effect size of 0.33, relevant to the assessment of known groups validity. The assessment of appropriate sample size for other measures of effect is considered in terms of precision, i.e., the width of the 95% Confidence Interval (CI). In terms of the assessment of convergent validity, a sample size of n = 300 would provide a precision of ±≈0.11 around a small Pearson’s correlation coefficient of 0.10, ±≈0.10 around a medium-sized correlation coefficient of 0.30, and ±≈0.09 around a large-sized correlation coefficient of 0.50 (Cohen, 1988; Cohen, 1992), allowing differentiation between such values. In terms of assessing test-retest reliability, the width of the intraclass correlation (ICC) CI would be ±0.05 around an ICC of 0.80 for a sample size of 200, ±0.065 for a sample size of 120, and ±0.1 for a sample size of 50 patients (Giraudeau and Mary, 2001; Shoukri et al, 2004). These estimates of precision thus suggest that, if the observed ICC was 0.80, an ICC of <0.70 could be ruled out even if only 50 patients reported no change between the two assessments.

**Criteria for inclusion and exclusion**

**Inclusion Criteria:**

1. Written informed consent prior to performance of any trial-related activity.
2. Adults ≥18 years of age (at the time of written consent).
3. Nocturia symptoms present for ≥6 months prior to Visit 1.
4. ≥2 nocturnal voids (an average over 3 days) as documented in the 3-day diary at the end of the Screening/Lifestyle changes period prior to Visit 2.
5. Nocturnal polyuria, defined as Nocturnal Polyuria Index (NPI) ≥33%, a ratio of nocturnal urine volume (NUV) in excess of 33% of total daily (24-hour) urine volume as documented in the diary period at the end of the Screening/Lifestyle changes period prior to Visit 2.
6. Bothered by nocturia as defined by a score of ≥3 on the Hsu 5-point Likert scale (moderate to extremely bothered) at Visit 2 (Hsu et al, Obstet Gynecol. 2014;125:35-43).
7. NI Diary Total Score of ≥20 points at Visit 2.
8. Additionally, to be randomized into the trial at Visit 4 subjects must respond to treatment defined as:

- ≥20% decrease in the nocturnal diuresis (that was recorded at Visit 2) as documented in the 3-day diary at the end of the active run-in prior to Visit 3.
- returning to at least 70% of the nocturnal diuresis (that was recorded at Visit 2) as documented in the 3-day diary at the end of the washout period prior to Visit 4.
- ≥2 nocturnal voids (an average over 3 days) as documented in the 3-day diary at the end of the washout period prior to Visit 4.

**Exclusion Criteria;**

1. Evidence of any significant voiding dysfunction resulting in abnormally low bladder capacity (defined as maximum voided volume [MUV] <250 mL) as documented in the 3-day diary at the end of the Screening/Lifestyle changes period prior to Visit 2.
2. History or evidence of obstructive sleep apnoea (as assessed by the Berlin questionnaire during screening).
3. History or diagnosis of any of the following urological diseases:
   - Interstitial cystitis (IC) or bladder pain disorder (BPS).
   - Chronic pelvic pain syndrome.
   - In men, subjects with suspicion of moderate or severe benign prostate hyperplasia (BPH) with or without bladder outlet obstruction (BOO) defined as IPSS ≥ 8 points and:
     - Urinary flow <5 mL/s as confirmed by uroflowmetry

or

- - - post-void residual volume (PVR) >150 mL as confirmed by bladder ultrasound performed.
  - Stress urinary incontinence or mixed incontinence, where stress incontinence is the predominant component based on prior history.
  - Moderate to severe daytime voiding dysfunction (e.g. overactive bladder, OAB) defined as:
- Urge urinary incontinence (an average of >1 episode /day in the 3-day diary period)

or

- Urgency (an average of >1 episode/day in the 3-day diary period)

or

- Frequency (an average of >8 daytime voids/day in the 3-day diary period).
  - 1. Surgical treatment, including transurethral resection, for BOO or BPH (in men) within the past 6 months prior to Visit 1.
    2. Genito-urinary tract pathology that can in the investigator’s opinion be responsible for urgency or urinary incontinence e.g., symptomatic or recurrent urinary tract infections, interstitial cystitis, bladder related pain, or stone in the bladder and urethra causing symptoms.
    3. Current or a history of urologic malignancies (e.g., bladder cancer), any lower urinary tract surgery, or previous pelvic irradiation.
    4. History of any neurological disease affecting bladder function or muscle strength (e.g., Multiple Sclerosis, Parkinson’s, spinal cord injury, spina bifida).
    5. Habitual or psychogenic polydipsia.
    6. Current uncontrolled hypertension.
    7. Serum triglyceride level >400 mg/dL.
    8. Current uncontrolled diabetes mellitus.
    9. Central or nephrogenic diabetes insipidus.
    10. Syndrome of inappropriate antidiuretic hormone.

14 History of :

- - Gastric retention.
  - Suspicion or evidence of cardiac failure.
  - Narrow-angle glaucoma.
  - Myasthenia gravis.
    - 1. Hyponatremia:
  - Serum sodium level <135 mmol/L at Visit 2 or Visit 3.
    - 1. Renal insufficiency:
  - Serum creatinine above normal limits and estimated glomerular filtration rate ≤60 mL/min.

1. Hepatic and/or biliary diseases:
   - Child-Pugh Class A, B, or C.
   - Aspartate aminotransferase and/or alanine aminotransferase levels ≥2x the upper limit of normal range.
   - Total bilirubin level >1.5 mg/dL.
2. Known or suspected hypersensitivity to desmopressin orally disintegrating tablets.
3. Pregnancy, breastfeeding, or a plan to become pregnant during the period of the trial. Subjects of reproductive age must have documentation of a reliable method of contraception. All pre- and perimenopausal subjects have to perform pregnancy tests. Amenorrhea of more than 12 months duration based on the reported date of the last menstrual period is sufficient documentation of post-menopausal status and does not require a pregnancy test.
4. Known alcohol or substance abuse.
5. Work or lifestyle that may interfere with regular night-time sleep e.g. shift workers.
6. Any other medical condition, laboratory abnormality, psychiatric condition, mental incapacity, or language barrier that, in the judgment of the Investigator, would impair participation in the trial.
7. Not sufficiently capable of completing electronic diary.
8. Previous treatment with desmopressin for nocturia.
9. Use of any prohibited therapy listed below:
   - Current or former (within 3 months prior to screening) treatment with any other investigational product.
   - Unstable electrostimulation or behavioural bladder training program less than 2 months prior to screening (stable electrostimulation or behavioural bladder training program started at least 2 months before screening are acceptable).
   - Thiazide diuretics.
   - Antiarrhythmic agents.
   - Potent cytochrome P450 (CYP) 3A4 isoenzyme inhibitors (e.g., protease-inhibitors, erythromycin, clarithromycin, fluconazole, ketoconazole, or itraconazole).
   - Botulinum toxin (cosmetic non-urological use is acceptable).
   - Valproate.

**Exit Interview**

The sample size for the Exit Interview was determined through power calculation (with desired power 0.80, and P<0.05) for a paired t-test assessing mean equivalence between Baseline and Week 12 for the NI Diary total score. Prior to stopping interviews, a planned interim analysis was performed to confirm adequate distribution of change on the NI Diary.

**Patient Global Impression Scales**

**Patient Global Impression of Severity Scale (PGI-S)**

Check the one number that best describes how your night time urination is now.

1. None
2. Mild
3. Moderate
4. Severe

**Patient Global Impression of Improvement Scale (PGI-I)**

Check the one number that best describes how your night time urination is now, compared with how it was before you began taking medication in this study.

- Very much better
- Much better
- A little better
- No change
- A little worse
- Much worse

The stem questions and response options for the PGI items included in the trial were based on feedback from a prior cognitive interview study that preceded inclusion in the clinical trial.

**Calculation of the Item Discrimination Index**

The item discrimination index was calculated based on the following steps:

1. Step 1: For each item, partition patients into 2 groups based on their item responses. The severe item responders consisted of those who responded either as “3 = quite a bit” or “4 = a great deal.”
2. Step 2: Partition patients into 2 groups based on their NI Diary total scores. The upper total and lower total groups consisted of the top and bottom 20% of respondents, respectively.
3. Step 3: For each item, calculate the proportion of those in the upper total group who were severe item responders (UpS); then, calculate the proportion of those in the lower total group who were severe item responders (LoS).
4. Step 4: Calculate the discrimination index based on severe item responses using the following formula:

UpS – LoS, where:

UpS = Proportion of upper total group who were severe item responders

LoS = Proportion of lower total group who were severe item responders

**Test-retest Reliability Calculation**

Test-retest reliability was computed for the three sub-samples of patients showing little or no change between Baseline and Week 1 (see Online Resource). 1) those who endorsed “No change” response on the PGI-I at week 1 (n=33); those who had no more than +/- 1 point of change between baseline and week 1 on the PGI-S (n=216); and those with no more than +/- 1 change in the average number of nocturnal voids between baseline and week 1 (n=125).

The ICC (2,1) was estimated as described Shrout and Fleiss by using the equation:

$$ICC (2,1)=\frac{BMS-EMS}{BMS+\left( k-1 \right)EMS+k(JMS-EMS)/n}$$

**Description of Anchor Variables**

Note: Uncollapsed anchor categories were defined a priori within the Psychometric Analysis Plan.

*Patient Global Impression-Severity (PGI-S)*

- Uncollapsed categories stratified patients based on the change in the PGI-S between Baseline and Week 12: -3, -2, -1, 0, 1, 2, 3.

Note: no patients reported a worsening of 3 points (ie, a score of 4).

*Patient Global Impression-Improvement (PGI-I)*

- Uncollapsed categories stratified patients based on the PGI-I rating at Week 12: “Very much better,” “Much better,” “A little better,” “No change,” “A little worse,” “Much worse,” or “Very much worse,”.

Note: no patients reported a worsening of “Very much worse”.

*3-Night Average Score on the NI Diary Q12 (NI Diary Q12)*

- Uncollapsed categories stratified patients based on the change in NI Diary Q12 between Baseline and Week 12: -4, -3, -2, -1, 0, 1, 2, 3, 4.

*3-Night Average Score (Nocturnal Void)*

- Uncollapsed categories stratified patients based on the change in the number of average nocturnal voids between Baseline and Week 12 into the following ranges: ≤‑3.5, >-3.5 to -2.5, >-2.5 to -1.5, >-1.5 to -0.5, >-0.5 to <0.5, ≥0.5

**Figure S2: Item Discrimination Curve Using Mean NI Diary Total Score at Baseline (Item 5 is presented in the main manuscript)**

**Stratified by Item 1: Difficult to concentrate**

|  |
| --- |

| **Stratified by Item 2: Low in energy and/or tired** |
| --- |

|  |
| --- |

| **Stratified by Item 3: Unable to be productive or complete daily activities** | |
| --- | --- |
|  |  |

|  |
| --- |

| **Stratified by Item 4: Avoid participating in activities** | |
| --- | --- |
|  |  |

|  |  |
| --- | --- |
|  | |

| **Stratified by Item 6: Limit your fluid intake** | |
| --- | --- |
|  |  |

|  |  |
| --- | --- |
|  | |

| **Stratified by Item 7: Lay awake after using the bathroom at night** | |
| --- | --- |
|  |  |

| **Stratified by Item 8: Worried about tripping or falling** |
| --- |

|  |  |
| --- | --- |
|  | |

| **Stratified by Item 9: Got too little sleep** |
| --- |

|  |
| --- |

| **Stratified by Item 10: Worry that the nocturia will get worse** |
| --- |

|  |
| --- |

| **Stratified by Item 11: Concerned with where the bathroom is** | |
| --- | --- |
|  |  |

|  |
| --- |

| **Stratified by Item 12: Does nocturia presently impact your life?** | |
| --- | --- |
|  |  |

Table S1: Within-subject change in NI Diary Total Score from Baseline to Week 12 using Nocturnal Voids as an Anchor

| Change in Nocturnal Void Anchor Category | Baseline Correlation | Change to Week 12 correlation | N | Mean (SD) | Median | Min, Max | 95% CI of Mean | P-Value^[1]^ | SES of Change^[2]^ | |
| --- | --- | --- | --- | --- | --- | --- | --- | --- | --- | --- |
| **Uncollapsed Categories** | 0.158 | 0.389 |  |  | |  |  |  | |  |
| ≤-3.5 |  |  | 9 | -34.3 (22.10) | -33.3 | -83,-7 | -51.25, -17.27 | 0.0016 | -1.55 | |
| >-3.5 to -2.5 |  |  | 40 | -37.5 (30.50) | -37.5 | -99,12 | -47.21, -27.70 | <.0001 | -1.23 | |
| >-2.5 to -1.5 |  |  | 83 | -22.0 (24.22) | -17.4 | -95,29 | -27.31, -16.74 | <.0001 | -0.91 | |
| >-1.5 to -0.5 |  |  | 73 | -14.7 (21.22) | -10.6 | -91,16 | -19.68, -9.78 | <.0001 | -0.69 | |
| >-0.5 to <0.5 |  |  | 21 | -8.0 (14.62) | -1.5 | -37,17 | -14.65, -1.34 | 0.0210 | -0.55 | |
| ≥0.5 (worsening) |  |  | 7 | 2.1 (14.32) | 0.0 | -15,22 | -11.19, 15.30 | 0.7171 | 0.14 | |

Abbreviations: Max = Maximum, Min = Minimum, NID = Nocturia Impact Diary, SD = Standard Deviation, SES = Standardized Effect Size

[1] The p-value for each individual change group is derived from a paired (within samples) t-test assessing the difference over time.
[2] Standardized Effect Sizes are calculated as the mean divided by the standard deviation. They are judged as: small = 0.20, moderate = 0.50, and large = 0.80.

Table S2: Within-subjects change in NI Diary Total Score from Baseline to Week 12 using the PGI-S

| Change in PGI-S  Anchor Category | Baseline Correlation | Change to Week 12 Correlation | N | Mean (SD) | Median | | Min, Max | | 95% CI of Mean | P-Value^[1]^ | SES of Change^[2]^ | |
| --- | --- | --- | --- | --- | --- | --- | --- | --- | --- | --- | --- | --- |
| **Uncollapsed Categories** | 0.573 | 0.669 |  |  | |  | |  |  | | |  |
| -3 |  |  | 24 | -60.6 (31.05) | -58.1 | | -99, 11 | | -73.75, -47.53 | <.0001 | -1.95 | |
| -2 |  |  | 51 | -35.8 (21.87) | -37.9 | | -81, 2 | | -41.93, -29.62 | <.0001 | -1.64 | |
| -1 |  |  | 100 | -17.4 (18.61) | -14.8 | | -73, 11 | | -21.13, -13.74 | <.0001 | -0.94 | |
| 0 |  |  | 59 | -6.0 (13.91) | -3.4 | | -48, 17 | | -9.65, -2.40 | 0.0015 | -0.43 | |
| 1 |  |  | 9 | 7.0 (12.20) | 0.0 | | -9, 29 | | -2.39, 16.36 | 0.1241 | 0.57 | |
| 2 |  |  | 1 | -22.7 (N/A) | -22.7 | | -23, -23 | | - | - | - | |
| 3 |  |  | 0 | - | - | | - | | - | - | - | |

Abbreviations: Max = Maximum, Min = Minimum, NID = Nocturia Impact Diary, SD = Standard Deviation, SES = Standardized Effect Size, PGI-S = Patient Global Impression – Severity
[1] The p-value for each individual change group is derived from a paired (within samples) *t*-test assessing the difference over time.
[2] Standardized Effect Sizes are calculated as the mean divided by the standard deviation. They are judged as: small = 0.20, moderate = 0.50, and large = 0.80.

Table S3: Within-groups change in NI Diary Total Score from Baseline to Week 12 using the PGI-I

| PGI-I  Anchor Category | Baseline Correlation | Change to Week 12 Correlation | N | Mean (SD) | Median | | Min, Max | | 95% CI of Mean | | P-Value^[1]^ | SES of Change^[2]^ | |
| --- | --- | --- | --- | --- | --- | --- | --- | --- | --- | --- | --- | --- | --- |
| **Uncollapsed Categories** | - | 0.639 |  |  | |  | |  | |  | | |  |
| Very Much Better |  |  | 90 | -38.8 (27.17) | -37.5 | | -99, 11 | | -44.47, -33.08 | | <.0001 | -1.43 | |
| Much Better |  |  | 74 | -20.4 (20.92) | -16.7 | | -81, 11 | | -25.23, -15.54 | | <.0001 | -0.97 | |
| A Little Better |  |  | 46 | -8.0 (15.34) | -5.9 | | -48, 29 | | -12.55, -3.44 | | 0.0010 | -0.52 | |
| No Change |  |  | 33 | -1.5 (10.39) | -0.8 | | -24, 17 | | -5.15, 2.22 | | 0.4227 | -0.14 | |
| A Little Worse |  |  | 4 | -6.6 (20.87) | -0.2 | | -35, 9 | | -39.83, 26.58 | | 0.5704 | -0.32 | |
| Much Worse |  |  | 3 | 8.0 (12.38) | 3.4 | | -2, 22 | | -22.81, 38.72 | | 0.3817 | 0.64 | |
| Very Much Worse |  |  | 0 | - | - | | - | | - | | - | - | |

Abbreviations: Max = Maximum, Min = Minimum, NID = Nocturia Impact Diary, SD = Standard Deviation, SES = Standardized Effect Size, PGI-S = Patient Global Impression – Severity
[1] The p-value for each individual change group is derived from a paired (within samples) *t*-test assessing the difference over time.
[2] Standardized Effect Sizes are calculated as the mean divided by the standard deviation. They are judged as: small = 0.20, moderate = 0.50, and large = 0.80.

Table S4: Within-groups change in NI Diary Total Score from Baseline to Exit Interview using the PGI-I (Exit Interview)

| PGI-I (EI)  Anchor Category | Baseline Correlation | Exit Interview Correlation | N | Mean (SD) | Median | | Min, Max | | 95% CI of Mean | | P-Value^[1]^ | SES of Change^[2]^ | |
| --- | --- | --- | --- | --- | --- | --- | --- | --- | --- | --- | --- | --- | --- |
| **Uncollapsed Categories** | - | 0.540 |  |  | |  | |  | |  | | |  |
| Very Much Better |  |  | 23 | -22.2 (14.94) | -23.5 | | -56, -1 | | -28.69, -15.77 | | <.0001 | -1.49 | |
| Much Better |  |  | 17 | -21.9 (16.61) | -21.2 | | -48, 4 | | -30.40, -13.32 | | <.0001 | -1.32 | |
| A Little Better |  |  | 10 | -8.2 (14.02) | -5.9 | | -37, 8 | | -18.21, 1.85 | | 0.0981 | -0.58 | |
| No Change |  |  | 13 | 0.8 (10.85) | 0.0 | | -27, 17 | | -5.80, 7.31 | | 0.8054 | 0.07 | |
| A Little Worse |  |  | 0 | - | - | | - | | - | | - | - | |
| Much Worse |  |  | 0 | - | - | | - | | - | | - | - | |
| Very Much Worse |  |  | 0 | - | - | | - | | - | | - | - | |

Abbreviations: Max = Maximum, Min = Minimum, NID = Nocturia Impact Diary, SD = Standard Deviation, SES = Standardized Effect Size.
[1] The p-value for each individual change group is derived from a paired (within samples) t-test assessing the difference over time.
[2] Standardized Effect Sizes are calculated as the mean divided by the standard deviation. They are judged as: small = 0.20, moderate = 0.50, and large = 0.80.

Table S5: Within-groups change in NI Diary Total Score from Baseline to Week 12 using the NI Diary Q12

| Change in NI Diary Q12 Anchor Category | Baseline Correlation | Change to Week 12 Correlation | N | Mean (SD) | Median | | Min, Max | | 95% CI of Mean | P-Value^[1]^ | SES of Change^[2]^ | |
| --- | --- | --- | --- | --- | --- | --- | --- | --- | --- | --- | --- | --- |
| **Uncollapsed Categories** | 0.816 | 0.858 |  |  | |  | |  |  | | |  |
| -4 |  |  | 16 | -78.9 (16.58) | -84.1 | | -99, -53 | | -87.74, -70.07 | <.0001 | -4.76 | |
| -3 |  |  | 27 | -53.2 (20.62) | -56.8 | | -92, -14 | | -61.31, -45.00 | <.0001 | -2.58 | |
| -2 |  |  | 35 | -38.5 (12.53) | -40.2 | | -65, -17 | | -42.78, -34.17 | <.0001 | -3.07 | |
| -1 |  |  | 67 | -18.7 (13.81) | -18.9 | | -51, 7 | | -22.06, -15.33 | <.0001 | -1.35 | |
| 0 |  |  | 98 | -7.3 (11.32) | -5.3 | | -38, 16 | | -9.55, -5.01 | <.0001 | -0.64 | |
| 1 |  |  | 19 | 6.0 (11.97) | 7.6 | | -24, 29 | | 0.25, 11.79 | 0.0417 | 0.50 | |
| 2 |  |  | 3 | 9.5 (7.08) | 10.6 | | 2, 16 | | -8.11, 27.05 | 0.1463 | 1.34 | |
| 3 |  |  | 0 | - | - | | - | | - | - | - | |
| 4 |  |  | 0 | - | - | | - | | - | - | - | |

Abbreviations: Max = Maximum, Min = Minimum, NID = Nocturia Impact Diary, SD = Standard Deviation, SES = Standardized Effect Size.
[1] The p-value for each individual change group is derived from a paired (within samples) t-test assessing the difference over time.
[2] Standardized Effect Sizes are calculated as the mean divided by the standard deviation. They are judged as: small = 0.20, moderate = 0.50, and large = 0.80.

Table S6: Within-subject change in NI Diary Total Score “1-Category” Anchor Improvement Groups

| Anchor | Change Level | N | Mean (SD) | Median | 95% CI of Mean | P-Value^[1]^ | SES of Change^[2]^ |
| --- | --- | --- | --- | --- | --- | --- | --- |
| Nocturnal Voids | >-1.5 to -0.5 | 73 | -14.7 (21.22) | -10.6 | -19.68, -9.78 | <.0001 | -0.69 |
| PGI-S | 1-Point  Improvement (-1) | 100 | -17.4 (18.61) | -14.8 | -21.13, -13.74 | <.0001 | -0.94 |
| PGI-I | A Little Better | 46 | -8.0 (15.34) | -5.9 | -12.55, -3.44 | 0.0010 | -0.52 |
| PGI-I Interview | A Little Better | 10 | -8.2 (14.02) | -5.9 | -18.21, 1.85 | 0.0981 | -0.58 |
| NI Diary Q12 | 1-Category  Improvement (-1) | 67 | -18.7 (13.81) | -18.9 | -22.06, -15.33 | <.0001 | -1.35 |

Abbreviations: Max = Maximum, Min = Minimum, NID = Nocturia Impact Diary, SD = Standard Deviation, SES = standardized effect size; PGI-I - Patient Global Impression – Improvement; PGI-S = Patient Global Impression – Severity

[1] The p-value for each individual change group is derived from a paired (within samples) t-test assessing the difference over time.
[2] Standardized Effect Sizes are calculated as the mean divided by the standard deviation. They are judged as: small = 0.20, moderate = 0.50, and large = 0.80.

Figure S3: Blinded CDF of the Change from Baseline in NI Diary Total Score at Week 12 for Anchors

| Anchor | Uncollapsed Categories at Week 12 |
| --- | --- |
| **Nocturnal Voids** |  |
| **PGI-I** |  |
| **PGI-I**  **Exit Interview** |  |

| PGI-S |  |
| --- | --- |

| NI Diary Q12 |  |
| --- | --- |

Abbreviations: CDF = cumulative distribution function; NI = nocturia impact

Figure S4: Receiver Operating Characteristic (ROC) Curve of NI Diary Change Score from Baseline to Week 12 Based on Attaining the Cut-Off of 1.0 Reduction in the Mean Number of Nocturnal Voids


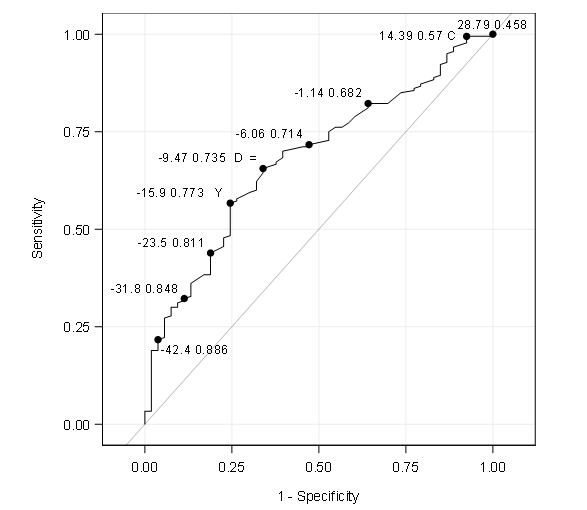


**Optimal Cut-Points Estimations For the NI Diary Change Score:**

Distance to 0,1 (indicated by “D” on the graph) = -9.47

Sens-Spec (indicated by “=” on the graph) = -9.47

Youden Index (indicated by “Y” on the graph) = -15.9

Average of the three estimates reported above = -11.6

**Exit Interviews: Additional Information**

The Exit Interview study was performed by a qualitative research team within Clinical Outcomes Solutions. The 66 exit interviews were completed approximately 6 months prior to the clinical trial final data lock – thus all data analyses was independent of full trial results. Trial data for the 66 exit interview participants was provided (Week 12 PGI-S, PGI-I, and nocuturnal voids). The PGI-I was asked again at the time of the interview and became the PGI-Interview anchor within the manuscript. Only the NI Diary and the nocturnal voids questions were queried for meaningfulness as these were considered key targets at the time. The NI Diary Q12, PGI-S, and PGI-I were debriefed during the interview, but participants were not asked about meaningfulness.

Table S7. Meaningful Improvement in Nocturnal Voids by PGI-I Response Category

| **Nocturnal Void MCT** | **Very Much Better**  **(n = 24)** | **Much Better**  **(n = 18)** | **A Little Better**  **(n = 11)** | **No Change**  **(n = 13)** | **Total**  **(N = 66)** |
| --- | --- | --- | --- | --- | --- |
| MCT = 1 | 12 | 10 | 6 | 2 | 30 |
| MCT = 2 | 9 | 5 | 1 | 1 | 16 |
| MCT = 3 | 2 | 3 | 0 | 0 | 5 |
| MCT = 4 | 1 | 0 | 0 | 0 | 1 |
| N/A- MCT probing not continued* | 0 | 0 | 2 | 1 | 3 |
| N/A- no change** | 0 | 0 | 2 | 9 | 11 |

*These include participants who experienced a reduction of one nocturnal void per night, who said this was not meaningful and were not probed further.

**These include participants who had no change in nocturnal urinations per night who therefore could not be probed on what constitutes a meaningful change.

Nearly half of the sample (n = 30) reported that a 1-void reduction was meaningful (Table 7S) with the majority of those (n = 22) stating that this would represent change in condition as “much better” or “very much better”. A total of 16 participants stated that a reduction of 2-voids would be meaningful, the majority of whom (n = 14) stated this would represent a change in condition that was “much better” or “very much better”.

A total of n = 52 participants therefore gave their interpretation on what constitutes a meaningful reduction in nocturnal voids. Stratifying these participants by level of improvement, a clear pattern emerged showing that participants who experienced a higher level of improvement in the PGI-I over the trial experienced a larger reduction in nocturnal voids per night. The average reduction in the PGI-I “very much better”, “much better”, “a little better”, and “no change” groups were 2.92 (SD = 1.18), 2.71 (SD = 1.86), 1.18 (SD = 0.75), and 0.23 voids (SD = 0.60), respectively.
